# Multicultural ideology among Chilean municipal workers: the role of prejudice and intercultural sensitivity

**DOI:** 10.3389/fpsyg.2025.1610421

**Published:** 2025-09-15

**Authors:** Gonzalo Martínez-Zelaya, María José Mera-Lemp, Florencia Guglielmetti-Serrano, Marian Bilbao

**Affiliations:** ^1^Faculty of Legal, Social and Educational Sciences, University of Viña del Mar, Viña del Mar, Chile; ^2^Escuela de Psicología, Universidad Gabriela Mistral, Santiago, Chile; ^3^Facultad de Psicología, Universidad Alberto Hurtado, Santiago, Chile

**Keywords:** municipal workers, immigration, multiculturalism, prejudice, intercultural sensitivity

## Abstract

**Introduction:**

Municipal employees are key actors in managing intercultural interactions in increasingly diverse public service settings. This study examined the relationships between prejudice, intercultural sensitivity, and multicultural ideology among Chilean municipal workers.

**Methods:**

A total of 197 participants completed an online questionnaire that included validated measures of these constructs, along with sociodemographic variables such as gender, age, educational level, religiosity, and contact with immigrant users.

**Results:**

A structural equation model was estimated using the DWLS estimator for ordinal data. The model showed good fit indices (CFI = 0.996; TLI = 0.996; RMSEA = 0.021; SRMR = 0.086). The results revealed that prejudice negatively predicted all dimensions of intercultural sensitivity, which in turn positively predicted support for cultural diversity and negatively predicted support for intercultural homogeneity. Female gender, higher educational level, and more frequent contact with immigrants were associated with lower levels of prejudice.

**Discussion:**

These findings highlight the mediating role of intercultural sensitivity in the relationship between underlying prejudicial attitudes and ideological support for multiculturalism. The study contributes to the theoretical understanding of affective pathways toward acceptance of diversity and offers practical implications for the design of training and policies that promote inclusive attitudes among municipal employees.

## 1 Introduction

The socioeconomic and political transformations experienced by Latin America and the Caribbean throughout the last decades have defined a new migration pattern for Chile, becoming one of the central receiving countries of intraregional immigrants (Organización Internacional del Trabajo, 2023). According to official data (Instituto Nacional de Estadísticas and Departamento de Extranjería y Migración, 2022), in 2002, immigrants represented 1.2% of Chile’s population, whereas in 2022, they accounted for 8.9% of its inhabitants. In this context, 92.9% of immigrants come from other countries of Latin America, mainly Venezuela (32.9%), Peru (15.4%), Colombia (11.7%), Haiti (11.4%), and Bolivia (9.1%).

The rise of immigration in Chile presents significant challenges, especially demanding that State institutions build the capacities to appropriately receive and welcome the immigrant population, satisfy their needs, and create proper conditions so they can develop their lives optimally within national borders (Colmenares and Thayer, 2022; Galaz Valderrama et al., 2017). This is a relevant point, as the available evidence indicates the material living conditions of this population tend to be precarious (CASEN, 2022), presenting important obstacles to regularizing their immigration status (Castro Zumarán et al., 2023), and in the access to social services, education, and health (Aninat and Vergara, 2019; Cabieses and Oyarte, 2020; Galaz Valderrama et al., 2017; Grau-Rengifo et al., 2021).

Although the state’s migration policies play a crucial role in Chile, local governments have a significant part in managing public services that support the development of citizens’ lives, creating the conditions for the exercise of their rights and satisfying their needs (Thayer Correa and Durán Migliardi, 2015). Furthermore, local territories are the stage on which relationships between immigrants and members of the majority society are configured, so municipalities’ actions are also relevant for managing intercultural coexistence (Thayer Correa et al., 2013).

In this regard, public workers, who operate in various areas such as education, health, and social services, become the first line of response for immigrants’ needs, intervening in the intergroup relationships between nationals and foreigners. This everyday response is framed within the “street-level bureaucracy” (Lipsky, 2010), where this type of work directly interacts with the population.

It is central to note that municipal street-level workers are characterized by having considerable discretionary power and authority to address the individual situations they encounter in their work (Lipsky, 2010; Maynard-Moody and Portillo, 2010). Even though this discretion can be a valuable tool for adapting to individuals’ specific needs, it can also lead to decision-making being influenced by biases and prejudices that workers develop regarding citizens (Maynard-Moody and Portillo, 2010; Tormann and Rapp, 2018; Gilson et al., 2014). When their attitudes toward citizens are positive, workers can mobilize resources and respond to their most urgent needs, even in the absence of proper public policies and in settings where resources are scarce (Schütze, 2019; Tartakovsky and Baltiansky, 2023; Thayer et al., 2020).

In the Chilean context, it has been observed that, along with the administrative barriers that hinder their inclusion, the immigrant populations tend to be targeted by discrimination by social services providers, which negatively impacts their possibilities to access the State’s programmatic offer (Galaz Valderrama et al., 2017). As pointed out by various authors (Carens, 2004; Colmenares and Thayer, 2022; Thayer et al., 2020), immigrants’ partial access to their rights is due, in part, to the type of social interactions they establish with those whoever make this access effective.

Likewise, municipal workers’ perceptions of the immigrant collective influence the degree to which they develop visions and ideologies that promote multicultural societies (Viola et al., 2018). Multiculturalism involves promoting positive evaluations of cultural diversity, supporting the idea that minority groups can fully and equitably participate in society, and accepting that all citizens, including members of cultural majorities, must adapt to one another (Berry et al., 1977). Municipal workers’ adherence to cultural pluralism is relevant because, as previously mentioned, they perform tasks that involve everyday contact with immigrant individuals (Bhuyan et al., 2024; Carrizales, 2019), and, in the case of Chile, this has increased exponentially in recent years.

Literature in this scope has also highlighted the role of municipal workers’ intercultural competencies (Carrizales, 2019; Hergüner, 2021; Bakar et al., 2015; Sousa and Almeida, 2016). These competencies include aspects such as the development of skills to communicate in a culturally sensitive manner with others, respecting differences, and paying attention to their interlocutor, which would facilitate involvement in communication and, in consequence, greater satisfaction and confidence in intercultural interactions with users (Chen and Starosta, 2000). Furthermore, this type of intercultural competence depends significantly on municipal workers’ attitudes, beliefs, and perceptions about immigrants, which can either hinder or promote their development (Akca and Ayaz-Alkaya, 2023; Bennett, 1986; Dollwet and Reichard, 2014; Mera-Lemp et al., 2020).

Since intercultural competencies facilitate the establishment of positive relationships with foreign populations, their development would result in better attitudes toward multiculturalism and, through this, favor interactions between municipal workers and citizens (Bhuyan et al., 2024; Chen and Starosta, 2000). This would enhance workers’ disposition to promote the exercise of immigrant people’s rights.

### 1.1 Multicultural ideology

Multiculturalism is frequently understood as an ideological stance that values the acknowledgment and appreciation of ethnic and cultural diversity (Berry, 1984; Morrison et al., 2010). It has been promoted as a strategy to enhance social cohesion and promote fairness among groups, underlying the acknowledgment of the dignity, rights, and inherent value of marginalized groups as fundamental to fostering social justice and equity (Fowers and Richardson, 1996). At the same time, literature has identified three different conceptualizations of multiculturalism (Guimond et al., 2014). The first one is linked to the sociodemographic presence of many groups with diverse geographical and ethnic origins. The second one is related to the legislation that promotes cultural heterogeneity, recognizing and improving the social status of immigrant groups and sanctioning discrimination against them. A multicultural approach entails recognizing intergroup differences, valuing cultural diversity, and affirming the identities of minority populations. This means implementing public policies to guarantee minority groups’ access to exercise rights to the same extent as the majority population. Within the field of social psychology, studies suggest that endorsing multicultural principles can positively influence intergroup relationships by encouraging respect for differences, reducing bias, and fostering more inclusive attitudes (Guimond et al., 2014).

On the other hand, the third definition of multiculturalism refers to a psychological perspective that promotes individuals’ positive attitudes toward points of view and ideologies that foster multicultural societies (van de Vijver et al., 2008). Thus, the adherence of host societies’ members to ideologies that encourage multiculturalism affects the relationships between the majority and minority groups significantly. This implies supporting immigrants in maintaining their cultures of origin while also conveying the need to accommodate diversity equitably (Arends-Tóth and van de Vijver, 2003). Although the endorsement of multiculturalism by members of the receiving countries appears crucial for achieving positive intergroup relations, its promotion may also prompt resistance, especially under conditions of high social dominance orientation and realistic or symbolic threat perceptions (Morrison et al., 2010; Sirlopú and Van Oudenhoven, 2013; Stephan et al., 2005; Urbiola et al., 2018; Vedder et al., 2017; Velasco González et al., 2008).

Support for multicultural ideology is crucial in the case of municipal workers, influencing the way they implement public policies to manage cultural diversity. The better their attitude toward the inclusion of immigrant collectives, the better they will be able to carry out actions that promote the inclusion of these groups (Tartakovsky and Baltiansky, 2023).

Studies that inquire about the antecedents of attitudes toward multiculturalism have shown that they are negatively associated with the perception of recently arrived foreigners as competitors for material or symbolic resources (Abreu Rivera and Piatkowska, 2024; Callens et al., 2019; Zhou et al., 2019). Although this line of research is incipient in Chile, it has been observed that, in the case of teachers—who, who, like municipal workers, serve as intermediaries in the implementation of public policy with immigrant populations—their degree of prejudice toward immigrants partly explains their attitudes toward multiculturalism in school contexts (Mera-Lemp et al., 2020).

### 1.2 Municipal workers’ prejudices

Prejudice is defined as a negative cognitive or affective response to outgroups’ members, playing a crucial role in the creation or maintenance of hierarchical intergroup relationships (Dovidio et al., 2010; Yzerbyt and Demoulin, 2010). Literature differentiates between types of prejudice (Pettigrew and Meertens, 1995; Stanke et al., 2024), proposing that blatant prejudice consists of beliefs that reject specific groups and direct contact with their members. On the other hand, subtle prejudice is characterized by a more distant and indirect attitude, linked to the defense of national values, exaggeration of cultural differences, and the denial of positive emotions toward members of the outgroup (Pettigrew and Meertens, 1995; Stanke et al., 2024).

These attitudes among members of host societies influence their support for various immigration policies proposed by the State, as well as the treatment accorded to immigrant individuals, and affect the degree of social cohesion or conflict present in different territories (Esses, 2021; Kende et al., 2021). In other fields of work, such as teaching, it has been demonstrated that teachers’ attitudes toward multiculturalism and levels of prejudice do not differ significantly from those of the general population (Mera-Lemp et al., 2024). It could be hypothesized that, in the case of municipal workers, these attitudes would significantly impact the way they interact and deliver services to the immigrant population, either positively or negatively influencing the wellbeing and integration of this group (Raaphorst and Groeneveld, 2018; Schütze, 2019; Tartakovsky and Baltiansky, 2023). Furthermore, the literature has demonstrated higher levels of racial prejudice can be related to lower levels of perceived cultural competence (Castillo et al., 2007), so it could be proposed that municipal workers’ beliefs could be affecting their skills when providing their services to immigrants.

Various theoretical models have consistently supported the notion that prejudice against immigrants is a significant predictor of Multicultural Ideology. According to Intergroup Threat Theory (Stephan and Stephan, 2000), perceived threats—both symbolic and realistic—provoke defensive responses to cultural diversity, thereby weakening support for multicultural approaches (Stephan et al., 2009). Similarly, Social Dominance Theory posits that individuals with a strong preference for hierarchical social structures, who tend to exhibit higher levels of prejudice toward subordinate groups such as immigrants, are more likely to reject multicultural models as they are perceived to undermine the social status quo (Sidanius and Pratto, 1999). Furthermore, intergroup ideological frameworks have shown that those with elevated levels of prejudice display lower endorsement of multicultural ideologies and a greater inclination toward assimilationist or monocultural models (Verkuyten, 2005; Yogeeswaran and Dasgupta, 2014). Empirically, recent studies have demonstrated that anti-immigration sentiments robustly predict opposition to diversity policies and limited recognition of the cultural contributions of migrant groups (Plaut et al., 2011). Taken together, these perspectives illustrate how prejudice against immigrants not only shapes interpersonal relations, but also directly influences the broader evaluation of multicultural social contexts.

In Chile, research on the values and beliefs of municipal workers regarding immigrant users is scarce. However, literature on the local general population’s attitudes reveals conflicts in the relationship between foreigners and members of Chilean society. This would be associated with an intensification of prejudice, linked to the idea that the presence of immigrants limits the access of Chileans to the job market, and the perception that immigration has given way to a rise in crime, augmenting social conflict and making Chile a worse place to live (Encuesta Nacional Bicentenario, 2023; Irarrázaval and Casielles, 2019; Mera-Lemp et al., 2019; Mera-Lemp et al., 2021a; Mera-Lemp et al., 2021b).

Likewise, recent studies about Chilean general population’s prejudice toward immigrant groups, report the presence of both blatant and subtle prejudices (Cárdenas et al., 2011; Tejeda Muñoz, 2018), moderate levels of affective prejudice (Carmona-Halty et al., 2019), and low social closeness perceptions (Arancibia and Cárdenas, 2022). There are no available antecedents about municipal workers’ prejudices toward immigrant users in our national context. Nevertheless, studies conducted with other types of municipal servants, such as municipal health workers, have suggested that their therapeutic relationship with immigrant patients depends on their level of acceptance, prejudice, and stereotypes held about this population, showing difficulties in implementing intercultural approaches due to these negative beliefs (Abarca-Brown, 2024; Cruz-Riveros et al., 2022; Tijoux and Ambiado, 2023).

Furthermore, in the case of Chilean teachers working at public schools, studies have shown that their attitudes toward immigrant people do not differ from those held by the general population (Mera-Lemp et al., 2021a; Mera-Lemp et al., 2021b). This has been attributed to how their social identities would be equally threatened by the presence of immigrants (Tajfel et al., 1979), mobilizing negative attitudes toward multiculturalism, even in school settings (Horenczyk and Tatar, 2002; Siqués et al., 2009; Vecina Merchante, 2006), which could be replicated in the case of municipal workers.

### 1.3 The role of intercultural sensitivity

In the context of public institutions, intercultural competency is linked to integrating and transforming knowledge about users and minority groups into standards, policies, practices, and attitudes, fostering service quality improvement (Hergüner, 2021). This supposes that front-line workers understand and respond to differences in respectful and sensitive manners, learning to adjust their behavioral patterns and interacting effectively with people of diverse cultural origins (Rice, 1997; Swann, 2012). Thus, several studies have suggested that the greater the intercultural competency shown by municipal workers, the better the treatment they provide to users, enhancing immigrants’ satisfaction with services, and fostering their adherence to government programs (Constantine, 2002; Fuertes et al., 2006; Saha et al., 2013; Wing and Sue, 2008).

In this context, competencies to successfully communicate in culturally diverse settings become a key resource for fostering better relationships between institutions and their beneficiaries, ensuring that services delivered meet their needs efficiently and effectively (Cram and Alkadry, 2018; Kim et al., 2024). Chen and Starosta (2000) define intercultural communication competence as a broad construct that encompasses the cognitive, emotional, and behavioral capacities individuals employ when engaging in intercultural interactions. Intercultural sensitivity is defined as the emotional dimension of intercultural communication, which encourages the awareness of respect and attention to cultural differences, adjusting one’s behavior. This implies the experience of positive emotions during interaction, and, consequently, confidence in one’s ability to successfully communicate in culturally diverse settings (Bennett, 1986; Chen and Starosta, 1998, 2000).

Research has suggested that the development of intercultural sensitivity depends on the degree to which individuals present prejudice and stereotypes toward minorities and their tendency to justify inequalities between social groups (Alexandra, 2018). Furthermore, denying diversity and ethnocentrism would also noticeably undermine this competence (Bennett, 1986; Hammer et al., 2003).

Besides, intercultural sensitivity could affect the degree to which individuals adhere to ideologies and attitudes that favor multiculturalism (Akcaoğlu and Kayiş, 2021; Kim and Connelly, 2019). This competence would promote both the recognition and affirmation of cultural differences, as well as the rejection of perspectives that devalue cultures of non-belonging (Bhawuk et al., 2008; Kim and Van Dyne, 2012). Even though intercultural sensitivity has been scarcely studied at the Chilean context, some studies have suggested that intercultural competences are a precursor to the extent to which members of the majority society want immigrants to maintain their cultural identities of origin and, at the same time, incorporate elements of the local culture (Martínez-Zelaya et al., 2020; Mera-Lemp et al., 2021a; Mera-Lemp et al., 2021b).

Based on this background, this study aims to evaluate the relationships between prejudice toward Latin American immigrants, intercultural sensitivity, and multicultural ideology, among municipal workers in Chile. As a hypothesis, we expect that: 1. The variance in Support for Multicultural Ideology will be predicted by prejudice; 2. The variance in Support for Multicultural Ideology will be predicted by Intercultural Sensitivity; 3. Intercultural sensitivity will mediate the relationship between prejudice and multicultural ideology.

## 2 Materials and methods

### 2.1 Study design

A quantitative, non-experimental, and cross-sectional design was used. A survey was conducted to measure levels of prejudice, intercultural sensitivity, and attitudes toward multiculturalism among municipal workers from the central zone of Chile.

### 2.2 Participants

Participants were 322 workers (37.1% men and 62.9% women) from five municipalities in central Chile. The average age was 42.8 years (*SD* = 11.9), with an average of 10.6 years (*SD* = 10.4) working at the municipality and 6.6 years (*SD* = 7.6) in the same position. It is important to note that the level of direct interaction with immigrants varied significantly, as some participants interacted with the public while others did not. Thus, the range was 0 to 45 h per week, corresponding to a full-time work week. The average was 5.7 h (*SD* = 11.3), indicating that most participants did not have frequent public contact. 18.6% of participants indicated that they “never” or “almost never” had contact with immigrants in the work context, while only 19.9% said it occurred “always” or “almost always.”

### 2.3 Variables and instruments

#### 2.3.1 Sociodemographic characteristics

An *ad hoc* questionnaire was elaborated for the purposes of this study. This questionnaire included gender, if the participants were part of the LGBTQI+ community, educational level, adscriptions to any religious denominations, type of contract, years working in this current position and in this municipality. *Religiosity* was assessed using a single-item measure: “How religious do you consider yourself to be?,” rated on a Likert scale from 1 (Not religious at all) to 10 (Very religious). *Contact in private life with immigrants* was measured by asking participants to rate how frequently they interact with immigrant individuals in their personal life, on a scale from 1 (Never) to 5 (Very frequently).

#### 2.3.2 Prejudice

The subtle and blatant prejudice scale developed by Pettigrew and Meertens (1995), and adapted into Spanish by Rueda and Navas (1996), was used. This scale assesses the levels of prejudice held by the host society toward a target population. The original scale consists of 20 items divided into two subscales. For the purposes of this study, a shortened version comprising 10 items was used, retaining the two original dimensions of the scale, but including only 5 items per dimension. The model fit indices were satisfactory [*CFI* = 0.997; *TLI* = 0.996; *SRMR* = 0.045; *RMSEA* = 0.057 (0.037–0.077)]. The reliability of the dimensions was adequate: subtle prejudice (*ordinal α* = 0.851; *ω* = 0.826) and blatant prejudice (*ordinal α* = 0.887; *ω* = 0.857). The overall scale demonstrated adequate reliability (ordinal *α* = 0.927; *ω* = 0.910).

#### 2.3.3 Attitudes toward multiculturalism

The Multicultural Ideology Scale (Spanish version by Sirlopú et al., 2015, based on Arends-Tóth and Van de Vijver, 2003), comprising 10 Likert-type items on a 7-point scale (1 = Strongly Disagree, 7 = Strongly Agree), was used. It assesses two dimensions: support for cultural diversity and support for homogeneity, which adopts a critical stance toward cultural diversity, questioning its value or implications. The model fit indices were satisfactory [*CFI* = 0.994; *TLI* = 0.993; *SRMR* = 0.048; *RMSEA* = 0.079 (0.062–0.097)]. The reliability for the overall scale (*ordinal α* = 0.913; *ω* = 0.900) and the dimensions Support for Diversity (*ordinal α* = 0.894; *ω* = 0.869) and Support for Homogeneity (*ordinal α* = 0.881; *ω* = 0.858) was adequate.

#### 2.3.4 Intercultural sensitivity

The version adapted and validated by Martínez-Zelaya et al. (2024) was used, which is based on the original version by Chen and Starosta (2000). This version was adapted and validated for Chilean municipal workers. It consists of 13 items and measures 4 dimensions. Items were rated on a scale from 1 (strongly disagree) to 5 (strongly agree). The model fit indices were adequate [*CFI* = 0.981; *TLI* = 0.975; *SRMR* = 0.074; *RMSEA* = 0.096 (0.083–0.109)]. The reliability of the scale dimensions was acceptable (Respect for Cultural Differences: *ordinal α* = 0.838, *ω* = 0.771; Interaction Confidence: *ordinal α* = 0.791, *ω* = 0.745; Interaction Enjoyment: *ordinal α* = 0.763, *ω* = 0.681; and Interaction Attentiveness: *ordinal α* = 0.701, *ω* = 0.678). Overall reliability was adequate (*ordinal α* = 0.908, *ω* = 0.882) (Table 1).

**Table 1:** Sample characterization.

Variable	Group	%
Gender	Male	36.1%
	Female	63.9%
LGTBIQ+ community	No	89.4%
	Yes	10.6%
Educational level	Secondary education	10.9%
	Technical and vocational education	17.7%
	University education	54.0%
	Postgraduate	17.4%
Belongs to a religious denomination	No	81.4%
	Yes	18.6%
Type of contract	Permanent contract	34.1%
	Fixed-term contract	24.4%
	Professional fees	29.7%
	Professional fees in social program	11.9%

Variable	*M*	*SD*	Min	Max
Age	43.52	11.98	18	67
Religiosity	4.90	2.95	1	10
Contact in private life	2.63	1.35	0	5
Contact in work life	2.41	1.39	0	5
Years in current position	6.58	7.59	0	40
Years working in municipality	10.38	10.45	0	43
Hours attending to immigrants	6.14	11.84	0	45

*N* = 322.

### 2.4 Procedure

The sample was obtained by inviting municipalities to participate in a study on the attitudes of Chilean municipal workers toward immigrants. The municipalities were selected based on two main criteria: the size of the municipality’s population and the percentage of residents born abroad. After selecting the municipalities and obtaining the necessary permissions, all city hall workers were invited to complete a survey. Participation was voluntary and anonymous through a web link distributed via internal municipal communication.

### 2.5 Data analysis

All analyses were conducted in R (v.4.4.3) using the packages lavaan, psych, semTools, semPlotas, haven, and MVN. Missing data were handled using the pairwise deletion method.

Prior to model estimation, we tested the assumptions required for structural equation modeling (SEM). The univariate item distributions were inspected to detect empty or low-frequency response categories. Multivariate normality was tested using Mardia’s test (Mardia, 1970), which indicated significant violations. Therefore, all ordinal variables were modeled using the Diagonally Weighted Least Squares (DWLS) estimator, which is robust to non-normality and suitable for ordinal data (Li, 2016a; Rhemtulla et al., 2012).

Multicollinearity was evaluated through correlation matrices and auxiliary linear regressions using factor scores. The correlation between subtle and blatant prejudice was excessively high (*r* = 0.99), suggesting collinearity issues. To address this, both dimensions were merged into a single latent construct, following Kline (2016) guidance on model parsimony and identifiability. Variance inflation factors (VIF) for all other observed predictors remained below five, indicating acceptable multicollinearity levels (Hair et al., 2019). No multivariate outliers were detected via Mahalanobis distance.

**Figure 1:**
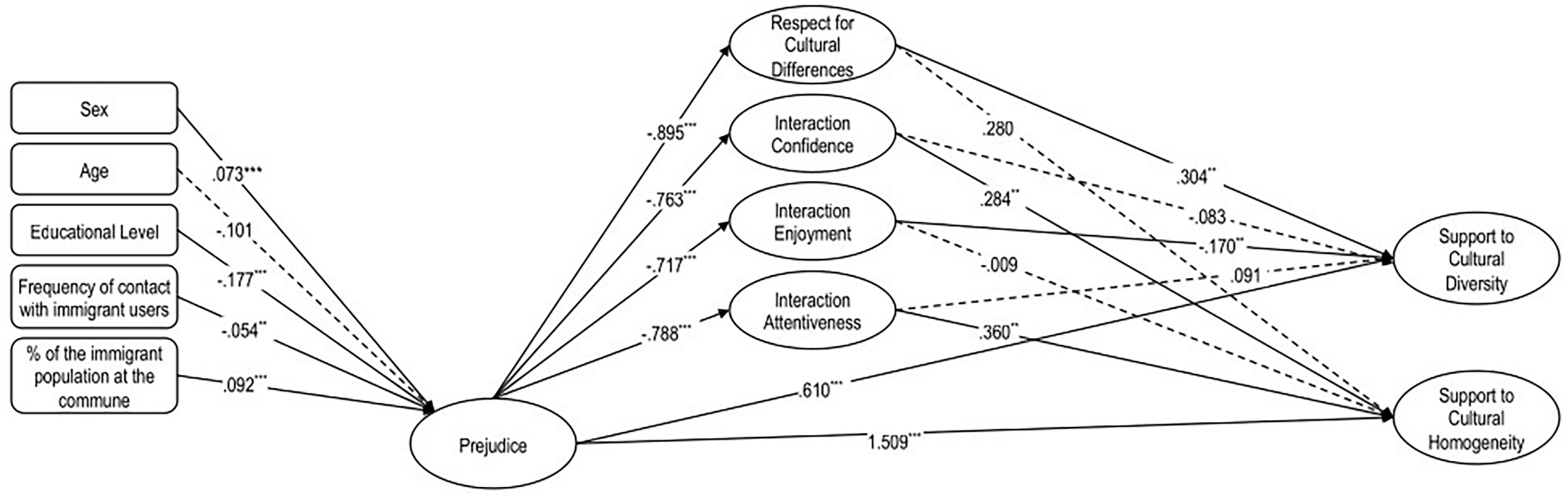
SEM diagram of the effect intercultural sensitivity dimensions on the relationship between prejudice and dimensions of attitudes toward multiculturalism.

Regarding control variables, sex, age, educational level, frequency of contact with immigrant users, and percentage of immigrant population in the municipality were included as observed exogenous predictors of prejudice in the SEM (see Figure 1). These variables were selected based on their documented relevance in prior literature (e.g., Arancibia Martini et al., 2016; Palmero Cámara et al., 2017), as well as their statistically significant associations with key model constructs in preliminary analyses (see Table 2). Their inclusion aimed to adjust for potential confounding effects and improve the explanatory power of the model.

**Table 2:** Descriptives of the studied variables.

Variable	*M*	*SD*	*α*	Min	Max
Prejudice					
Prejudice	2.40	0.857	0.927	1	5
Intercultural sensitivity					
Respect for cultural differences	4.35	0.628	0.838	1	5
Interaction confidence	3.98	0.775	0.792	1	5
Interaction enjoyment	4.36	0.715	0.764	1	5
Interaction attentiveness	4.07	0.675	0.701	1	5
Attitudes toward multiculturalism					
Support to cultural diversity	5.65	1.07	0.894	1	7
Support to cultural homogeneity	2.49	1.36	0.881	1	7

### 2.6 Ethical considerations

The research followed all necessary ethical procedures for respecting human rights in studies involving people, as outlined in international agreements. All participants took part voluntarily and were duly informed about the purpose and characteristics of the study, as well as the possibility of withdrawing at any time without justification. Participants gave their consent by reading an informed consent form, ensuring confidentiality and anonymity, before completing the evaluation instruments. The data were treated confidentially, and any elements that could reveal participants’ identities were removed. The research project was approved by the Scientific Ethics Committee of the university affiliation of the first author.

## 3 Results

The results (see Table 2) show that prejudice is present in the sample at low to moderate levels. Due to the high collinearity between Subtle Prejudice and Blatant Prejudice (*r* = 0.99), both constructs were merged into a single latent variable referred to as *prejudice*.

On the other hand, attitudes toward multiculturalism (*M* = 5.60, *SD* = 1.104) indicate moderate to high levels, suggesting that the sample holds an open ideology toward multiculturalism. Similarly, Intercultural Sensitivity (*M =* 4.21; *SD =* 0.549) also shows moderate to high levels among the sample of workers.

Correlation analyses between sociodemographic variables and the studied variables (see Table 3), show that sex was linked with enjoyment through intercultural communication, indicating that men tend to perceive higher levels of satisfaction in these interactions. Age positively correlated with confidence, and attention across intercultural communication exchanges.

**Table 3:** Correlations between sociodemographic variables and study variables.

Variable	Gender	Age	Educational level	Frequency of contact with immigrant users	% of the immigrant population at the commune
Prejudice					
Prejudice	0.114^***^	0.100^***^	−0.325^***^	0.031	−0.008
Intercultural sensitivity					
Respect for cultural differences	−0.007	0.049	0.074	0.085^*^	−0.062
Interaction confidence	−0.105^*^	0.160^**^	−0.074	0.154^***^	−0.103^*^
Interaction enjoyment	−0.174^**^	0.057	0.151^**^	0.046	0.029
Interaction attentiveness	−0.063	0.191^***^	−0.160^**^	0.244^***^	−0.156^**^
Attitudes toward multiculturalism					
Support for diversity	−0.072^*^	0.010	0.046	0.043	−0.119^***^
Support for homogeneity	0.108^**^	0.043	−0.161^***^	−0.050	−0.001

Statistical significance: ^*^p < 0.05; ^**^p < 0.01; ^***^p < 0.001.

Additionally, educational level was negatively associated with both types of prejudice. Participants’ educational background presented a negative relationship with attention in intercultural communication, but its association with enjoyment was positive. Also, educational level was negatively related to support for homogeneity. Besides, correlation analyses revealed that the more frequently participants have contact with immigrant users, the more they pay attention to cultural differences in intercultural communication, and their confidence in their capability to succeed in these interactions tends to increase. Furthermore, results show that the higher the percentage of immigrant population is in the communes where participants work, the lower their capability to pay attention and confidence to intercultural communication.

A correlational analysis was conducted to examine the relationship between the study variables in the model (see Table 4). Prejudice was strongly and negatively correlated with attitudes toward multiculturalism and with intercultural sensitivity. In contrast, intercultural sensitivity was positively associated with the dimension of support to cultural diversity. Furthermore, the dimension of support to cultural homogeneity was negatively associated with intercultural sensitivity.

**Table 4:** Correlations between prejudice, intercultural sensitivity, and their dimensions.

Variable	1	2	3	4	5	6	7
						
Prejudice							
1. Prejudice	–						
Intercultural sensitivity							
2. Respect for cultural differences	−0.895^***^	–					
3. Interaction confidence	−0.763^***^	0.683^***^	–				
4. Interaction enjoyment	−0.717^***^	0.642^***^	0.547^***^	–			
5. Interaction attentiveness	−0.788^***^	0.705^***^	0.602^***^	0.565^***^	–		
Attitudes toward multiculturalism							
6. Support to cultural diversity	−0.769^***^	0.749^***^	0.552^***^	0.469^***^	0.640^***^	–	
7. Support to cultural homogeneity	0.765^***^	−0.629^***^	−0.466^***^	−0.554^***^	−0.467^***^	−0.732^***^	–

Statistical significance: ^*^p < 0.05; ^**^p < 0.01; ^***^p < 0.001.

To determine which components of Intercultural Sensitivity best account for the indirect effect through this variable, a structural equation model (SEM) was conducted, incorporating the four dimensions of Intercultural Sensitivity as mediating variables in the relationship between prejudice and the dimensions of attitudes toward multiculturalism (Figure 1).

This analysis accounted for 62.9% of the variance in Support for Cultural Diversity, with a significant direct association (*β* = −0.610; *p* < 0.001), and a significant total association (*β* = −0.769; *p* < 0.001). These results indicate the presence of a mediation effect between prejudice and Support for Cultural Diversity through the dimensions of Intercultural Sensitivity. In this relationship, indirect associations were observed through Respect for Cultural Differences (*β* = −0.272; *p* < 0.001) and Interaction Enjoyment (*β* = 0.122; *p* = 0.016).

On the other hand, the analysis accounted for 68.4% of the variance in Support for Homogeneity, revealing a significant and positive direct association (*β* = 1.509; *p* < 0.001) and a significant total association as well (*β* = 0.765; *p* < 0.001). These findings indicate a mediation effect between prejudice and Support for Homogeneity via the dimensions of Intercultural Sensitivity. Indirect associations were observed through Interaction Attentiveness (*β* = −0.283; *p* = 0.006) and Interaction Confidence (*β* = −0.217; *p* = 0.004) (Table 5).

**Table 5:** Direct and Indirect associations of SEM.

Effects through…	**β**	SE	*p*
Direct			
Prejudice—respect for cultural differences	−0.895	0.209	0.000
Prejudice—interaction confidence	−0.763	0.075	0.000
Prejudice—interaction enjoyment	−0.717	0.067	0.000
Prejudice—interaction attentiveness	−0.788	0.107	0.000
Respect for cultural differences—support to cultural diversity	0.304	0.084	0.008
Interaction confidence—support to cultural diversity	−0.083	0.068	0.198
Interaction enjoyment—support to cultural diversity	−0.170	0.075	0.009
Interaction attentiveness—support to cultural diversity	0.091	0.073	0.204
Prejudice—support to cultural diversity	−0.610	0.289	0.001
Respect for cultural differences—support to cultural homogeneity	0.280	0.122	0.068
Interaction confidence—support to cultural homogeneity	0.284	0.103	0.001
Interaction enjoyment—support to cultural homogeneity	−0.009	0.073	0.879
Interaction attentiveness—support to cultural homogeneity	0.360	0.125	0.002
Prejudice—support to cultural homogeneity	1.509	0.571	0.000
Indirect			
Prejudice—respect for cultural differences—support to cultural diversity	−0.272	0.168	0.009
Prejudice—interaction confidence—Support to cultural diversity	0.063	0.082	0.212
Prejudice—interaction enjoyment—support to cultural diversity	0.122	0.082	0.016
Prejudice—interaction attentiveness—support to cultural diversity	−0.072	0.090	0.197
Prejudice—respect for cultural differences—support to cultural homogeneity	−0.251	0.262	0.096
Prejudice—interaction confidence—support to cultural homogeneity	−0.217	0.130	0.004
prejudice—interaction enjoyment—support to cultural homogeneity	0.007	0.077	0.878
Prejudice—interaction attentiveness—support to cultural homogeneity	−0.283	0.180	0.006

Respect for cultural differences *R*^2^ = 0.801. Interaction confidence *R*^2^ = 0.583. Interaction enjoyment *R*^2^ = 0.514. Interaction attentiveness *R*^2^ = 0.621. Support to cultural diversity *R*^2^ = 0.629. Support to cultural homogeneity *R*^2^ = 0.684.

## 4 Discussion

The present study investigated the associations among prejudice, intercultural sensitivity, and attitudes toward multiculturalism in a sample of Chilean municipal employees. Findings indicate that participants exhibited generally low to moderate levels of overall prejudice. Specifically, realistic prejudice was reported at a modest level, whereas subtle prejudice emerged at an intermediate level. This pattern suggests a somewhat negative appraisal of cultural differences, which may be shaping the nature of interactions between municipal personnel and immigrant communities. These results align with previous research conducted among Chilean teachers and university students, which has consistently observed a predominance of subtle over overt forms of prejudice, despite the use of alternative measures to assess prejudice (Mera-Lemp et al., 2021a; Mera-Lemp et al., 2021b).

Conversely, levels of intercultural sensitivity were found to be moderately high. This suggests that participants tend to develop capabilities that facilitate intercultural communication and positive emotional experiences associated with such interactions. Also, their approaches toward ideologies and perspectives that support multicultural societies tend to be positive. However, these attitudes do not reflect an unreserved openness to cultural diversity. Taken together, findings suggest that municipal workers show a positive perspective regarding their interactions with immigrant service users. This outlook may contribute to more positive contact experiences and improve the quality-of-service delivery.

### 4.1 The role of sociodemographic variables

Regarding educational level, participants with higher education tended to report lower levels of prejudice and greater enjoyment in intercultural interactions. This is consistent with prior findings suggesting that education fosters cognitive complexity, tolerance for ambiguity, and internalization of egalitarian values (Peri, 2018; Arancibia Martini et al., 2016).

Gender differences were also observed, particularly in the enjoyment in intercultural interactions, where men reported slightly higher scores. This may reflect gendered patterns in emotional expression and social interaction styles, with men in professional settings possibly perceiving intercultural exchanges as less threatening or more novel. However, no significant gender differences were found in support for multicultural ideology, suggesting that ideological orientations may be less susceptible to gendered patterns than affective responses.

Regarding the relationships between sociodemographic variables and the studied variables, gender was found to correlate significantly and positively with blatant prejudice, indicating that women perceive higher levels of prejudice compared to men. Additionally, a significant negative relationship was observed between gender and the enjoyment dimension of intercultural sensitivity, suggesting that being male is associated with greater enjoyment in communication with immigrant individuals. These findings contrast with those reported by other studies, which have found that men tend to exhibit more negative attitudes toward members of minority groups (Dozo, 2015). However, they seem to be consistent with recent research in Chile, which has shown that women report higher levels of insecurity in areas where immigrant populations reside, compared to men (Encuesta Nacional Bicentenario, 2023). It is worth noting that attitudes toward multicultural ideology were not significantly related to the participants’ gender.

On the other hand, age was positively and significantly correlated with blatant prejudice, consistent with previous studies that have reported a link between increasing age and more negative attitudes toward individuals perceived as different. This has been associated with lower cognitive flexibility and reduced openness to change as individuals progress through the life cycle (Palmero Cámara et al., 2017). However, the results also indicate that older participants exhibited greater confidence and attention during interactions. This could be attributed to the greater experience that older workers possess in interacting with service users, which may enhance their expectations regarding the outcomes of these interactions. Participants’ ages showed no significant relationships with support for multicultural ideology.

Additionally, educational level was negatively associated with both types of prejudice. At the same time, its relationship with the enjoyment of interaction was positive, indicating that higher levels of education are linked to more favorable attitudes toward immigrant individuals. Also, highly educated workers showed greater enjoyment across intercultural communication, which their low levels of prejudice could prompt. These findings are consistent with existing literature, which has systematically shown the relationship between these variables (Arancibia Martini et al., 2016; Peri, 2018). In contrast, it was found that individuals with lower educational levels paid more attention during intercultural communication interactions. As we mentioned, these participants tend to show higher levels of prejudice. Thus, hypothetically, it is possible that negative feelings and concerns about immigrant users could lead to a higher awareness and focus on cultural differences, prompting attentiveness in these types of encounters. We also found that workers with lower formal education support cultural homogeneity to a greater extent than those with higher education, presenting concerns about diversity, as previous studies have suggested (Sirlopú et al., 2015).

Furthermore, our results show that the larger the immigrant population in the communes where participants work, the lower their attentiveness through intercultural communication, and, moreover, their support for cultural diversity decreased. Literature has suggested that the actual or perceived size of the non-native population can have an important influence on host society members’ attitudes toward the newcomers, augmenting the perception of them as competitors for different kinds of material, social, and symbolic resources (Kawalerowicz, 2021; Steele and Perkins, 2019; Stephan et al., 2005). This could be particularly salient for municipal workers who must facilitate immigrants’ access to services and opportunities within the state programs, negatively affecting their decision-making processes and the quality of their assistance to this population.

Overall, these results highlight the importance of paying attention to female and lower-skilled workers, who seem to reject immigrant users to a greater extent, to reduce their levels of prejudice and enhance their capabilities to communicate with different others. Also, it is important to consider the situation of workers who provide services in territories with a high concentration of immigrants, who seem to be less prone to support diversity, and are highly exposed to intercultural contact through their everyday labor. These considerations are relevant because of these workers’ role in applying public policies and the impact of their discretionary power in delivering resources (Maynard-Moody and Portillo, 2010; Tormann and Rapp, 2018; Gilson et al., 2014).

### 4.2 The relationship between prejudice, intercultural sensitivity, and multicultural ideology

As expected, prejudice exhibited significant negative associations with all components of intercultural sensitivity, with the strongest correlations observed with the dimensions of respect for differences and enjoyment in interaction, followed by trust and attention. Additionally, prejudice was strongly correlated with attitudes toward multiculturalism. Regarding intercultural sensitivity, all its dimensions were positively and significantly associated with attitudes toward multiculturalism. Specifically, the dimension of respect for differences exhibited the highest correlation with this variable, followed by attention in interaction, enjoyment, and trust.

Thus, these results suggest that individuals who exhibit lower negative evaluations of immigrant populations tend to be more sensitive in intercultural communication and are more likely to hold positive attitudes toward multiculturalism. These findings are consistent with previous studies conducted with general Chilean populations and teachers from the same country (Martínez-Zelaya et al., 2020; Mera-Lemp et al., 2024).

The SEM model reveals that prejudice has strong associations with both supporting cultural diversity and promoting cultural homogeneity. This finding is relevant because it highlights the importance of considering that municipal workers’ attitudes toward immigrants are related to their willingness to adopt approaches that could benefit this population or obstruct their access to resources. Prejudice is associated with their support for immigrants’ exercise of rights, and potentially, it could hinder their capacity to develop culturally responsive practices. Again, the relevance of their beliefs and valuations about immigrant users is related to their position as agents who must apply public policies, embodying the role of the State (Lipsky, 2010; Raaphorst and Groeneveld, 2018; Schütze, 2019; Tartakovsky and Baltiansky, 2023).

Moreover, part of the association of prejudice with attitudes toward multiculturalism can be related to different facets of intercultural sensitivity, which, in turn, facilitates the development of multicultural ideology. More specifically, respect for cultural differences seems to be a key factor that mediates the effect of prejudice on support for cultural diversity. Also, enjoyment through interactions plays a role as a mediator between these two variables. These outcomes stress the development of recognition and legitimization of immigrant users’ cultural backgrounds, needs, and rights, to improve municipal workers’ attitudes (Raaphorst and Groeneveld, 2018; Schütze, 2019; Tartakovsky and Baltiansky, 2023).

On the other hand, according to the model, prejudice had an indirect association with support for cultural homogeneity through the dimensions of confidence and attentiveness in interactions, showing positive relationships between these variables. A possible reason for these results could be that high levels of confidence could be linked to the tendency to overlook or render invisible cultural differences, and to pay greater attention to individual aspects in intercultural interactions. It seems to be consistent with the literature on acculturation processes in Chileans as receiving society members, which emphasizes the prominence of an individualistic approach toward the immigrant population, focusing on individual characteristics and obscuring aspects of cultural belonging. This could be associated with greater support for a homogenizing perspective that stresses the application of standard policies and actions for the entire population, without considering the particularities of the immigrant condition (Mera-Lemp et al., 2021a; Mera-Lemp et al., 2021b; Thayer Correa et al., 2013). Nevertheless, this might be further investigated in future research.

However, decreasing prejudice and considering the components of intercultural sensitivity can be important assets in improving municipal workers’ use of their discretionary faculties. Considering that public policies that benefit the immigrant population settled in Chile are limited (Colmenares and Thayer, 2022), and that mainstream society members perceive social and economic competition (Encuesta Nacional Bicentenario, 2023; Irarrázaval and Casielles, 2019; Mera-Lemp et al., 2021a; Mera-Lemp et al., 2021b), this could greatly help guarantee equal treatment and justice in their access to resources provided by the State.

It is worth noting that an alternative structural model—where multicultural ideology predicts intercultural sensitivity, which in turn influences levels of prejudice—was proposed by one of the reviewers. While we acknowledge the conceptual plausibility of this directionality, we chose not to test it in the present study due to both theoretical and methodological considerations. From a theoretical standpoint, previous literature has consistently positioned prejudice as an antecedent of both intercultural attitudes and ideological orientations (Pettigrew and Meertens, 1995; Stephan and Stephan, 2000; Verkuyten, 2005). Moreover, intercultural sensitivity is commonly understood as an emotional-communicative competence that is shaped by individuals’ underlying biases and beliefs, rather than as a driver of those beliefs (Bennett, 1986; Chen and Starosta, 2000). From a methodological perspective, testing the alternative model would have increased model complexity and risked overfitting, given the relatively limited sample size. Nonetheless, future studies with larger and more diverse samples may benefit from examining different causal paths, including reciprocal or bidirectional models.

In sum, this study contributes theoretically by empirically validating a structural model that links prejudice, intercultural sensitivity, and multicultural ideology among municipal employees—a group strategically positioned in the frontline of public service provision in diverse societies. By integrating these constructs, the study advances the understanding of how socio-cognitive and affective variables interact in shaping inclusive attitudes. Practically, the findings underscore the need for targeted training programs that not only inform employees about diversity, but also cultivate emotional readiness and enjoyment in intercultural encounters. Interventions focused on reducing prejudice and enhancing intercultural sensitivity may foster stronger commitment to multicultural values and improve institutional responsiveness. At the policy level, the study highlights the importance of embedding intercultural competencies into public service frameworks, especially in municipalities with rising immigrant populations. Enhancing the capacity of local governments to promote inclusive, culturally responsive services is not only a matter of administrative efficiency, but a key pillar of democratic coexistence and social cohesion in plural societies.

### 4.3 Limitations, strengths, and future research lines

This study presents several limitations that warrant acknowledgment. First, the use of a cross-sectional design for measuring the variables restricts the ability to infer causal relationships regarding the mechanisms underlying attitudes toward multiculturalism. Second, the relatively small sample size, comprising only 322 municipal employees from five municipalities in central Chile, limits the generalizability of the findings. This limitation stems from the specificity of the target population and the challenges associated with accessing these workers, primarily due to bureaucratic constraints within their institutions.

One of the methodological limitations of this study relates to the high collinearity observed between the dimensions of prejudice. Although it was originally planned to analyze the dimensions of subtle prejudice and overt prejudice separately, both showed a correlation close to unity, indicating significant empirical overlap between the constructs. This collinearity can affect the stability of estimates and generate identification problems in structural equation models (Kline, 2016). For this reason, we opted to use a composite measure of prejudice, integrating both dimensions into a single latent construct. This decision allowed us to maintain the parsimony of the model and avoid the problems associated with severe multicollinearity, although it also limited the possibility of analyzing the specific mechanisms associated with each form of prejudice separately.

Although the use of the Diagonally Weighted Least Squares (DWLS) estimator has been widely recommended for structural equation models with ordinal variables (Li, 2016b; Rhemtulla et al., 2012), its application has certain methodological limitations. In this study, DWLS was used considering the ordinal nature of the data and a sample size of 322 participants, a figure that falls within the range considered acceptable for this type of estimation. However, it should be noted that the performance of DWLS is sensitive to sample size: when N is less than 200, it can generate biased estimates and unstable standard errors (Flora and Curran, 2004). Furthermore, this estimator assumes that ordinal variables reflect continuous and normally distributed latent variables, which is rarely fully verified in applied data (Forero et al., 2009). Another critical aspect is that DWLS requires the estimation of a polychoric correlation matrix, which can become numerically unstable when there are categories with extremely low or empty frequencies (Savalei, 2010). Finally, unlike robust methods such as the MLR (Maximum Likelihood Robust) estimator, DWLS does not allow direct estimation under the assumption of ignorable missing data, which may limit its applicability in studies with incomplete data (Rosseel, 2012).

Additionally, the study did not incorporate measures assessing the behavioral dimension of intercultural sensitivity. Other relevant variables, such as the frequency of intergroup contact, perceived threat, or stereotypes, were also not considered, which may have provided a more comprehensive understanding of attitudes toward immigrant groups. Future research should aim to include larger and more geographically diverse samples of municipalities. Incorporating additional variables that may influence attitudes toward multiculturalism would also enhance the depth and applicability of subsequent findings.

Nevertheless, this research contributes to understanding municipal workers’ approaches to immigrant populations, which entails new and complex challenges for everyday interactions, how discretionary authority is used, and the duty to carry out national policies upholding the principles underpinning them. Also, it suggests interesting implications for interventions, which could include prejudice reduction and the training of intercultural communication, considering some key elements of work design, such as skills variety, task significance, and feedback (Sánchez-Hernández et al., 2020). This may be beneficial not only to immigrant users, but also to promote municipal employees’ wellbeing at their workplace, by facilitating positive experiences through their everyday tasks. As some studies have suggested (Gutentag et al., 2018; Muir et al., 2022), serving culturally diverse users could turn out to be a stressor when workers have no proper training. Thus, it is relevant to consider the prevention of job exhaustion as well as tensions between municipal workers and immigrant users.

These could be relevant in South–South migration contexts, which demand that public institutions with limited experience in these areas develop specific policies, services, and practices in new multicultural settings.

## Data Availability

The raw data supporting the conclusions of this article will be made available by the authors, without undue reservation.
